# Associations between Zinc Deficiency and Metabolic Abnormalities in Patients with Chronic Liver Disease

**DOI:** 10.3390/nu10010088

**Published:** 2018-01-14

**Authors:** Takashi Himoto, Tsutomu Masaki

**Affiliations:** 1 Department of Medical Technology, Kagawa Prefectural University of Health Sciences, 281-1, Hara, Mure-Cho, Takamatsu, Kagawa 761-0123, Japan; 2 Department of Gastroenterology and Neurology, Kagawa University School of Medicine, Kagawa 761-0123, Japan; tmasaki@med.kagawa-u.ac.jp

**Keywords:** zinc deficiency, HCV-related chronic liver disease, nonalcoholic steatohepatitis, liver cirrhosis, insulin resistance, hepatic steatosis, hepatic encephalopathy, iron overload, lipid peroxidation, insulin-like growth factor-1

## Abstract

Zinc (Zn) is an essential trace element which has favorable antioxidant, anti-inflammatory, and apoptotic effects. The liver mainly plays a crucial role in maintaining systemic Zn homeostasis. Therefore, the occurrence of chronic liver diseases, such as chronic hepatitis, liver cirrhosis, or fatty liver, results in the impairment of Zn metabolism, and subsequently Zn deficiency. Zn deficiency causes plenty of metabolic abnormalities, including insulin resistance, hepatic steatosis and hepatic encephalopathy. Inversely, metabolic abnormalities like hypoalbuminemia in patients with liver cirrhosis often result in Zn deficiency. Recent studies have revealed the putative mechanisms by which Zn deficiency evokes a variety of metabolic abnormalities in chronic liver disease. Zn supplementation has shown beneficial effects on such metabolic abnormalities in experimental models and actual patients with chronic liver disease. This review summarizes the pathogenesis of metabolic abnormalities deriving from Zn deficiency and the favorable effects of Zn administration in patients with chronic liver disease. In addition, we also highlight the interactions between Zn and other trace elements, vitamins, amino acids, or hormones in such patients.

## 1. Introduction

Zinc (Zn) is an essential trace element that plays pivotal roles in cellular integrity and biological functions related to cell division, growth and development [1,2]. Zn acts as a cofactor for many enzymes and proteins involved in anti-oxidant, anti-inflammatory and apoptotic effects [3]. Zinc-binding proteins represent approximately 10% of human proteomes, with more than 300 enzymes having zinc ions within their catalytic domains [4]. Two types of Zn transporters that maintain Zn homeostasis are identified: ZnTs (zinc transporters) and ZIPs (Zrt-, Irt-like proteins) [5]. ZnTs lower cytoplasmic Zn by transporting Zn through cell-surface membranes and intracellular organelles, while ZIPs increase cytoplasmic Zn.

Zn homeostasis is primarily regulated in the liver. Therefore, chronic liver damage results in the impairment of zinc homeostasis, and eventually zinc deficiency [6,7,8,9,10,11]. Zn deficiency consequently initiates a variety of metabolic abnormalities, including insulin resistance, hepatic steatosis, iron overload and hepatic encephalopathy (HE) in patients with chronic liver disease. By contrast, a decrease in albumin synthesis leads to Zn deficiency in patients with liver cirrhosis [12]. Recent studies have revealed the putative mechanisms by which Zn deficiency evokes a variety of metabolic abnormalities in patients with fatty liver, chronic hepatitis, or liver cirrhosis. The interactions between Zn deficiency and these metabolic abnormalities are summarized in Table 1.

Here, we mainly focus on the metabolic abnormalities deriving from Zn deficiency in patients with chronic liver disease and the beneficial effects resulting from zinc administration in such patients. Finally, the interactions between Zn deficiency and levels of other trace elements, vitamins, amino acids, or hormones are described.

## 2. Association between Zn Metabolism and Its Metabolic Abnormality

### 2.1. Zn Deficiency and Liver Cirrhosis

Liver cirrhosis is considered the most advanced stage of chronic liver disease. An unfavorable hepatic reserve results in numerous metabolic disorders, such as hypoalbuminemia. Consequently, a relative increase in α2 macroglobulin, which more strongly binds to Zn [13], caused a substantial increase in the urinary excretion of Zn [14]. Moreover, Zn absorption from the small intestine was often impaired in patients with liver cirrhosis [15,19]. Such mechanisms result in severe Zn deficiency in patients with liver cirrhosis.

### 2.2. Zn Deficiency and Hepatic Encephalopathy

Hepatic encephalopathy (HE) is one of the most common neuropsychiatric complications in patients with liver cirrhosis. The pathogenesis of HE has not been completely understood, although ammonia is considered to play a key role. Approximately 90% of patients with liver cirrhosis and HE have elevated ammonia levels in the plasma [9]. HE in liver cirrhosis patients was associated with a high prevalence of Zn deficiency [20]. Poor Zn status resulted in the impairment of nitrogen metabolism by reducing the activity of urea cycle enzyme, ornithine transcarbamylase, in the liver [16,21] and of glutamine synthetase in the muscle [22]. Indeed, serum Zn levels were inversely correlated with blood ammonia levels in patients with liver cirrhosis [23].

### 2.3. Zn Deficiency and Hepatocellular Carcinoma

Hepatocellular carcinoma (HCC) is the fifth most common form of cancer worldwide [24]. Liver cirrhosis appears to be a decisive risk factor leading to the progression to HCC.

The roles of trace elements underlying hepatocarcinogenesis have not been fully understood yet, although previous studies have identified a number of signal transduction pathways that are involved in this process. Elevated serum copper (Cu) levels were associated with the development of HCC [25,26,27,28]. Hepatic Cu content was significantly higher in the HCC tissue than that in the surrounding liver parenchyma [29,30]. By contrast, Zn concentrations in HCC tissues were lower than those in the surrounding hepatic parenchyma [29,30]. Zn may be involved in the regulation of apoptosis in HCC cells [31]. Zn also has a possibility to downregulate hypoxia-inducible factor-1α (HIF-1α) in the malignant cells [32]. Therefore, Zn deficiency may account for the proliferation of HCC cells. However, no significant difference in serum Zn levels was apparent between HCC and liver cirrhosis patients [33,34].

Based on the observations of intracellular Zn concentrations in different carcinomas, it has been postulated that these changes in Zn levels may contribute to the development of tumors by affecting a wide variety of molecular structures, such as receptors, kinases, caspases, phosphatases and transcriptional factors [35].

The decisive role of Zn in malignant transformation may derive from change in the expression of Zn transporters such as Zip4, Zip6, Zip7 and Zip10, as shown by the previous studies on certain types of carcinomas [36,37,38]. Notably, the expression of Zip14 was decreased during the development of HCC, coupled with a reduction in intracellular Zn levels [39]. Zip14 localizes to the cell membrane of normal hepatocytes and is a functional transmembrane transporter involved in the uptake of zinc into the cell. Therefore, its downregulation may explain the decreased Zn levels in HCC cells. On the other hand, the expression of Zip 4 gene, which is associated with acrodermatitis enteropathica [40], was upregulated in human and mouse HCC tissues, compared with surrounding non-cancerous tissues [41]. Zip4 influenced the expression of matrix metalloproteinase (MMP)-2 and MMP-9, which are directly involved in angiogenesis and the degradation of basement membrane collagen, in the HCC cell lines [42]. MMP-2 and MMP-9 are in the family of zinc-containing enzymes [43]. Therefore, Zip4 may regulate the expression of MMP-2 and MMP-9 by influencing Zn concentration in the HCC tissues.

Serum Zn status may be recognized as a prognostic serological hallmark after initial hepatectomy in HCV-related HCC patients. Imai and colleagues elucidated that HCC patients with lower serum Zn levels at the preoperative stage had significantly lower overall survival than those with a normal range of Zn levels [44].

### 2.4. Zn Deficiency and HCV Infection

Hepatitis C virus (HCV) is known to induce a spectrum of chronic liver diseases from chronic hepatitis to liver cirrhosis, and ultimately to HCC. More than 184 million people worldwide are estimated to be infected with HCV worldwide at present [45]. Persistent HCV infection often evokes mitochondrial oxidative stress [46], and a variety of resulting metabolic abnormalities, including insulin resistance, dyslipidemia, iron overload and hepatic steatosis [47,48]. Zn has potential cytoprotective effects against oxidative stress, apoptosis and inflammation [49].

Zn deficiency may trigger oxidative stress in patients with HCV-related chronic hepatitis and liver cirrhosis, termed HCV-related chronic liver disease (CLD) [50]. A decrease in serum Zn levels was observed in patients with asymptomatic HCV-carrier as well as those with HCV-related CLD, compared with serum Zn levels in normal healthy controls [51]. However, no significant correlation was found between serum Zn levels and loads of HCV RNA in patients infected with HCV. These data may imply that HCV infection probably affects Zn metabolism, although HCV itself has no direct effect on serum Zn levels in those patients.

Previously, NS3 proteinase, which is involved in the process of HCV replication, proved to be a zinc-containing enzyme [52]. In addition, NS5A protein was considered a Zn metalloprotein [53]. These findings suggest that Zn may have inhibitory effects in the proliferation and replication of HCV.

### 2.5. Zn Deficiency in Nonalcoholic Steatohepatitis

Nonalcoholic fatty liver disease (NAFLD) is currently the most prevalent liver disease worldwide, characterized by the accumulation of triglycerides in the liver, and the absence of excessive alcohol consumption. NAFLD covers a spectrum of liver diseases that range from simple steatosis called nonalcoholic fatty liver (NAFL) through nonalcoholic steatohepatitis (NASH), which is associated with hepatic inflammation and fibrosis in addition to simple steatosis [54].

Previous studies elucidated that dietary habits are likely to affect Zn metabolism in patients with NAFLD. Lower oral intake of Zn was observed in patients with NAFLD than in normal healthy controls [55]. NASH patients had significantly lower oral intake of Zn than NAFL patients [56]. Zn deficiency in such patients may be due to this lower Zn intake. Zn deficiency results in mitochondrial oxidative stress and subsequently iron over load, insulin resistance, and hepatic steatosis in patients with NASH.

Zn and zinc transporters play crucial roles in the attenuation of endoplasmic reticulum (ER) stress-related signaling and the unfolded protein response (UPR) [57]. Therefore, Zn deficiency may potentially induce or exacerbate ER stress and apoptosis. Kim and colleagues recently elucidated that consumption of a zinc-deficient diet exacerbated ER-stress-induced apoptosis and hepatic steatosis in experimental mice models [58]. The authors proposed that Zip14 mediated Zn transport into hepatocytes to inhibit protein-tyrosine phosphatase 1B, which suppressed apoptosis and steatosis associated with ER stress [59].

### 2.6. Zn Deficiency and Insulin Resistance in Patients with HCV-Related CLD

Zn plays a pivotal role in the secretion and activation of insulin. Specifically, Zn participates as a potent physiological regulator of insulin signal transduction through its inhibitory effect on protein tyrosine phosphatase 1b, the key phosphatase that dephosphorylates the insulin receptor [60]. Therefore, it has been well known that Zn is absolutely indispensable for the regulation of glucose homeostasis. Zn deficiency seems to be associated with the pathogenesis of type 2 diabetes mellitus (DM), supporting the notion that Zn deficiency may result in the exacerbation of insulin resistance. Zn deficiency may derive from hyperzincuria in patients with type 2 DM [61].

Insulin resistance and/or concurrent type 2 DM are often associated with the occurrence of HCV-related CLD [62]. Previously, we elucidated that Zn deficiency caused the exacerbation of insulin resistance in patients with HCV-related CLD [17,63]. Zn deficiency may contribute to higher serum ferritin levels and lower IGF-1/IGFBP-3 ratios, surrogate measures for circulating free IGF-1 levels [64], and thus substantially exacerbate the insulin resistance in such patients [18].

Depressed serum Zn levels were found in some patients with NAFLD (unpublished data). Zn deficiency is likely to evoke insulin resistance in such patients by way of almost the same mechanism as chronic HCV infection, including hyperferritinemia and/or lower circulating free IGF-1 levels [65].

Zn deficiency is also observed in patients with primary biliary cholangitis (PBC), an autoimmune liver disease characterized by progressive destruction of the intrahepatic bile ducts, leading to cholestasis [66]. Serum Zn levels are gradually decreased as the hepatic fibrosis develops in those patients. Advanced-stage PBC patients also have higher insulin resistance, suggesting that Zn deficiency may exacerbate insulin resistance in such patients [67].

### 2.7. Zn Deficiency and Iron Overload in Patients with HCV-Related CLD

Persistent HCV infection and nonalcoholic fatty liver disease (NAFLD) are well recognized cofactors affecting body iron storage [48]. A patient’s serum ferritin level is ordinarily used as an indicator of iron storage in the liver. We previously showed an inverse correlation between serum Zn and ferritin levels in patients with HCV-related CLD, implying that Zn deficiency might result in iron overload in those patients [17,63].

It has been well established that Zn and iron may compete for access to transporters. Formigari and colleagues suggested that divalent metal transporter 1 (DMT1) is a possible candidate zinc-iron transporter [68]. It is of interest that Zn competed with iron uptake in cells overexpessing Zip14, implicating Zip14 as another possible candidate for transport of both Zn and iron in the liver [69].

### 2.8. Iron Overload and Insulin Resistance

Iron often influences glucose metabolism. The amount of iron storage in the body was found to be significantly associated with the development of glucose intolerance or type 2 DM in a general population [70].

In addition, iron overload reciprocally seems to affect insulin action. Iron storage interferes with the production of glucose by the liver. Hepatic extraction of insulin and the body’s metabolism of insulin is reduced with increasing iron storage, leading to peripheral hyperinsulinemia.

This status is called “iron overload-related insulin resistance” [71]. Iron overload-related insulin resistance was observed in CLD-C and NAFLD patients with hyperferritinemia, who are frequently associated with hepatic steatosis and/or fibrosis [48,72].

### 2.9. Decrease in Circulating Free IGF-1 Levels and Insulin Resistance in Patients with HCV-Related CLD

**Figure 1:**
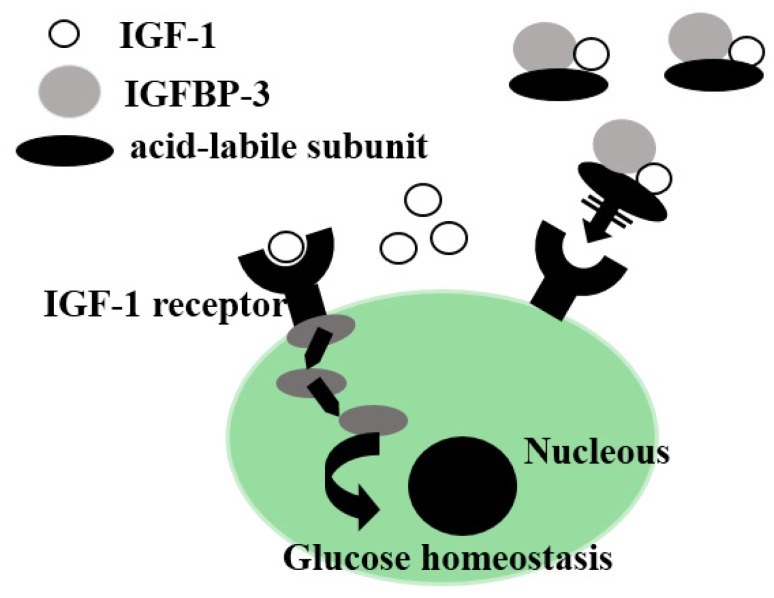
Putative mechanisms by which a decrease in free IGF-1 levels cause insulin resistance in patients with HCV-related CLD. IGF-1, insulin-like growth factor; IGFBP, IGF-binding protein; HCV, Hepatitis C virus; CLD, chronic liver disease.

Insulin-like growth factor-1 (IGF-1), a liver-derived humoral growth factor, has crucial anabolic and metabolic actions. It is secreted from hepatocytes and has a negative feedback control on growth hormone release from the pituitary gland. The biological activity of IGF-1 is primarily dependent on IGF-binding proteins (IGFBPs). The binding of IGFBP-3 to IGF-1 has been shown to inhibit the bioavailability of IGF-1 [73]. Therefore, free IGF-1 is considered the bioactive form of IGF-1 (Figure 1). However, free IGF-1 levels are difficult to determine directly. The ratio of IGF-1/IGFBP-3 is usually used as a surrogate estimate of free IGF-1 [64].

Zn is likely to participate in the stabilization of IGF-1 transcripts [74]. Thus, the IGF-1/IGFBP-3 ratio, a surrogate measure for circulating free IGF-1 level, had a positive correlation with serum Zn levels in patients with HCV-related CLD [18]. It is of interest that the IGF-1/IGFBP-3 ratio was inversely associated with the values of homeostasis model for assessment of insulin resistance (HOMA-IR), an indicator for insulin resistance, suggesting that a lower circulating free IGF-1 concentration might result in the exacerbation of insulin resistance in such patients.

### 2.10. Zn Deficiency and Hepatic Steatosis in Patients with HCV-Related CLD

A previous study demonstrated that Zn deficiency was linked to hepatic steatosis in an experimental animal model of fatty liver induced by tetracycline [75]. We confirmed that serum Zn levels were decreased as the degrees of hepatic steatosis developed in patients with HCV-related CLD [17]. Moreover, serum Zn levels were decreased in inverse proportion to the intensity of 4-hydroxy-2′-nonenal (4-HNE), an indicator for lipid peroxidation, in the liver of those patients [17].

Zn has been considered to participate in the enhancement of peroxisome proliferator-activated receptor-α (PPAR-α), a regulator of lipid homeostasis [76]. Zn is necessary for the DNA-binding activity of PPAR-α. Therefore, Zn deficiency may result in a decline of DNA-binding activity, and thereby facilitate lipid peroxidation, ultimately exacerbating hepatic steatosis.

**Figure 2:**
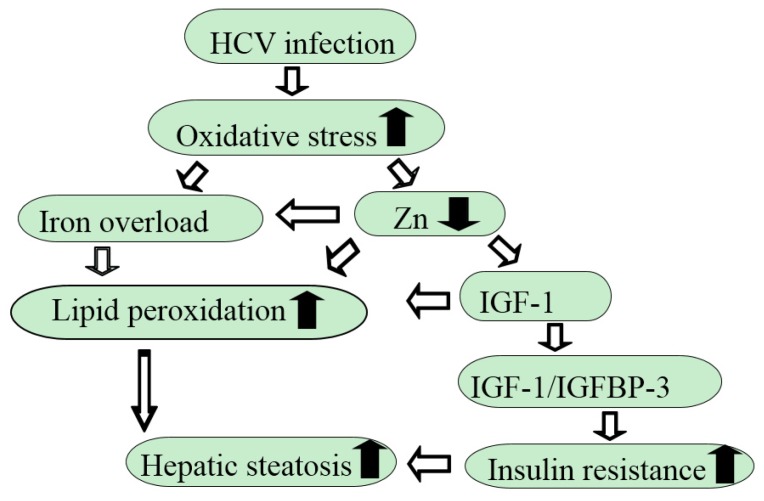
Putative mechanisms by which chronic HCV infection evokes insulin resistance and hepatic steatosis.

We also revealed that a lower IGF-1/IGFBP-3 ratio might cause more advanced hepatic steatosis in patients with HCV-related CLD [18]. A significant correlation was confirmed between insulin resistance and the severity of hepatic steatosis in those patients. Higher insulin resistance may result in a lower ratio of IGF-1/IGFBP-3 in HCV-related CLD patients with severe steatosis (Figure 2). The lower ratio of IGF-1/IGFBP-3 was also observed in patients with NAFLD [77], suggesting that a decrease in circulating free IGF-1 levels may account for exacerbation of insulin resistance and advanced hepatic steatosis.

Iron overload is also believed to cause lipid peroxidation, and subsequently to lead to hepatic steatosis in patients with HCV-related CLD [72]. We confirmed a significant correlation between the intensity of hepatic 4-HNE expression and serum ferritin levels in such patients [17].

### 2.11. Zn Metabolism and Wilson’s Disease

Wilson’s disease is an autosomal recessive, hereditary disorder of Cu metabolism due to defective Cu-transporting ATPase activity, leading to the accumulation of Cu in the liver and basal ganglia of the brain [78]. The accumulation of Cu may affect the metabolisms of other trace elements, including Zn and iron. However, few studies have focused on imbalances of trace elements in patients with Wilson’s disease. Ferenci and colleagues previously documented that slightly increased hepatic accumulation of Zn and iron were observed in such patients [79].

### 2.12. Zn Metabolism and Hemochromatosis

Patients with hereditary hemochromatosis, characterized by HFE point mutations [80], are more susceptible to iron overload when factors such as alcohol, HCV infection, and abnormal porphyrin metabolism are present [48]. Even in the absence of hereditary hemochromatosis, there are several conditions associated with secondary iron overload. A few studies have focused on the relationships between iron overload and other trace element statuses either in experimental models of hemochromatosis or in patients with hemochromatosis. Unfortunately, Zn status in patients with hemochromatosis remains controversial. Vayenas and colleagues elucidated that hepatic Zn content was increased in iron-overload Wister rats [81]. Adams and colleagues also revealed that hepatic zinc content was significantly higher in the livers of patients with hemochromatosis than that in normal livers [82]. The increase in hepatic Zn content might substantially derive from increased intestinal absorption of Zn and hepatic sequestration. To the contrary, Beckett and colleagues recently documented that iron overload did not have a significant effect on Zn status [83].

## 3. Therapeutic Effects of Zn Administration

Zinc administration has shown favorable effects on metabolic abnormalities in patients with chronic liver disease. The effects of zinc supplementation and possible mechanisms of these effects are summarized in Table 2, and described in detail below.

### 3.1. Attenuation of Hepatic Inflammation 

Zn has beneficial effects on hepatic inflammation in patients with HCV-related CLD. We previously documented that the administration of polaprezinc, a complex of Zn and l-carnosine, showed anti-inflammatory effects in the liver by attenuating hepatic iron storage in such patients [90]. However, the administration of zinc did not directly affect HCV viremia in our study, although Takagi and colleagues revealed that the combination therapy of zinc with interferon (IFN)-α was more effective against HCV eradication than IFN treatment alone [91]. Likewise, Zn administration also prevented an increase in serum alanine aminotransferase (ALT) levels of patients with chronic hepatitis C during IFN-based treatment [92].

### 3.2. Improvement of Insulin Resistance

Zn supplementation is likely to have favorable effects on impaired glucose tolerance. Yoshikawa and colleagues demonstrated that oral administration of Zn (II)-dithiocarbamate complex improved hyperglycemia, glucose intolerance and insulin resistance in an experimental animal model of type 2 DM [95]. It is of interest that an increase in adiponectin synthesis by the administration of Zn complexes resulted in the improvement of insulin resistance in those experimental animal models.

Oral Zn administrations, using zinc sulfate or zinc acetate, have been prescribed in patients with T2DM. A systemic review by Jayawardena documented that Zn supplementation had beneficial effects on glycemic control in such patients [96]. The authors speculated that the effect of Zn might be mediated by increasing IGF-1. Surprisingly, the authors revealed that Zn administration also improved dyslipidemia in T2DM patients. However, all these studies included dietary supplementation with other antioxidant vitamins and minerals together with Zn. Hence, it is difficult to conclude that the beneficial effects were due to Zn alone.

It is of interest that long-term Zn treatment improved impaired glucose tolerance in patients with advanced liver cirrhosis [89]. Further examinations will be required to clarify the efficacy of Zn administration in HCV-related CLD or NAFLD patients accompanied by insulin resistance.

### 3.3. Attenuation of Hepatic Steatosis

Sugino and colleagues investigated the effect of Zn (polaprezinc) in a mouse model of NASH. Zn supplementation did not affect the steatosis, but visibly attenuated fibrosis in the liver [97]. Another study documented that treatment with zinc sulfate reversed alcohol-induced steatosis in male mice via reactivation of hepatocyte nuclear factor-4α (HNF-4α) and PPAR-α [98]. 

Unfortunately, the effects of Zn administration on hepatic steatosis in patients with CLD-C or NAFLD has not been fully verified yet. Hence, Zn supplementation should be evaluated as an optional treatment for such patients [99].

### 3.4. Improvement of Hepatic Encephalopathy

Long-term Zn supplementation significantly improved the grade of HE and blood ammonia levels in patients with liver cirrhosis [84,85,86,87,88]. Zn administration in patients with liver cirrhosis and HE potentially resulted in the recovery of urea synthesis in the liver and glutamine synthesis in the muscle. Supplementation with branched-chain amino acids (BCAAs) enhances detoxification of ammonia from the skeletal muscles by the amidation process for glutamine synthesis [100]. Interestingly, it was found that a combination treatment with BCAAs and Zn decreased blood ammonia levels more than a treatment with BCAAs alone in patients with liver cirrhosis [101].

### 3.5. Inhibitory Effect on the Development of HCC

Zn administration appears to have a preventive effect on the development of HCC. Matsumura and colleagues documented that chronic hepatitis C patients treated with polaprezinc, a complex of zinc and l-carnosine, had a significantly lower incidence of HCC than those without administration of polaprezinc [94]. Maintenance of favorable hepatic reserve by Zn supplementation may account for the lower incidence of HCC.

Insulin resistance seems to be one of the risk factors for the development of HCC [102]. Zn supplementation may result in an improvement of insulin resistance in patients with HCV-related CLD or NASH. Accordingly, a treatment with Zn should be considered as a therapeutic strategy in such patients [103].

### 3.6. Reduced Cu Absorption from the Small Intestine

It has been widely established that oral Zn administration often provides Wilson’s disease patients with favorable effects. Zn administration induced the synthesis of metallothionein (MT), a copper-binding protein [104], in the small intestine and the liver. MT binds to newly absorbed Cu and prevents it from passing from the small intestine into the circulation. Some studies, using experimental animal models for Wilson’s disease, have also revealed an increase in hepatic MT synthesis, and sequestration of excess hepatic Cu content by the administration of Zn [105]. A significant correlation was found between MT and Zn concentrations in the duodenal mucosa of 15 patients with Wilson’s disease by the treatment with zinc sulfate or penicillamine [106]. Accordingly, Zn acetate has been approved for the treatment of Wilson’s disease, especially in early stage patients [93].

## 4. Interactions between Zinc and Other Trace Elements or Vitamins

It has been widely recognized that some trace elements, including Cu and iron, compete with Zn, while selenium and vitamin A are positively associated with Zn status. Table 3 shows interactions between Zn and other trace elements, vitamins, amino acids, or hormones status.

### 4.1. Zn and Cu

It is well known that Zn competes with Cu [8]. The risk of Cu deficiency in patients with continuously high dose of Zn prescription has been warranted [108,116]. High-dose Zn supplementation causes enterocytes to produce MT which binds to Zn. Whereas, Cu binds more avidly to MT than Zn, eventually leading to Cu deficiency [117]. We previously performed the additional treatment with polaprezinc (51mg/day of Zn for 6 months) in 14 patients with HCV-related CLD. However, none of the HCV-related CLD patients on this regimen suffered from Cu deficiency [90]. To the contrary, a lower intake of Zn was more effective than a moderately higher intake of Zn in inducing changes associated with a decreased Cu status in postmenopausal women [118].

The ratio of serum Cu to Zn levels has been utilized as a clinical assessment in certain diseases [119]. Among chronic liver diseases, elevated Cu/Zn ratios have been observed in patients with liver cirrhosis or HCC [33,34]. Therefore, the ratio of Cu/Zn can be monitored for estimating the severity of liver damage. However, the ratio cannot discriminate HCC patients from liver cirrhosis patients. It is of interest that elevation of the Cu/Zn ratio was observed in HCC patients alone among hepatobiliary cancer patients [107].

### 4.2. Zn and Iron

Zn also competes with iron. We previously showed a weakly inverse correlation between serum zinc and ferritin levels in patients with HCV-related chronic liver disease [17]. The inverse correlation between serum Zn and ferritin was also found in HCV-related CLD patients associated with NAFLD [109]. The putative mechanism is explained in the paragraph regarding “zinc deficiency and iron overload” in this review.

Interestingly, Zn supplementation caused an increased iron concentration in the duodenal mucosa of patients with Wilson’s disease, although the mechanism of this change remains unclear [106]. Further examinations will be required to clarify this phenomenon in such patients.

### 4.3. Zn and Selenium

We previously revealed that serum selenium levels were positively associated with serum Zn levels in patients with HCV-related CLD [111]. We also confirmed a positive correlation between serum selenium and albumin levels in such patients. Therefore, selenium deficiency might result in hypoalbuminemia, and subsequently cause Zn deficiency in those patients. Likewise, Thuluvath and Triger found a positive correlation between serum zinc and selenium levels among patients with various chronic liver diseases, including alcoholic liver disease, primary biliary cholangitis, cryptogenic cirrhosis, chronic active autoimmune hepatitis [110].

It is of interest that the administration of selenium modulated serum Zn levels in diabetic rats fed a zinc-deficient diet [120].

### 4.4. Zn and Vitamin A

A previous study elucidated that significant correlations between serum Zn and vitamin A or retinol-binding protein (RBP) levels were found in patients with liver cirrhosis [112]. Zn deficiency seems to impair the synthesis of RBP [7,121,122]. Therefore, the authors speculated that Zn deficiency might result in portosystemic shunting, and subsequent vitamin A deficiency by decreasing the release of RBP in such patients [112].

### 4.5. Zn and Vitamin D

Serum 25-hydroxy vitamin D (25-OH vitamin D) levels are also decreased in proportion to the severity of hepatic fibrosis as well as serum Zn levels in patients with HCV-related CLD [123]. Surprisingly, no significant correlation was found between serum 25-OH vitamin D and Zn levels in such patients [114], although serum Zn levels were significantly associated with serum 25-OH vitamin D levels among women with primary ovarian insufficiency [113].

### 4.6. Zinc and Amino Acids 

Moriyama and colleagues documented that there was not a significant correlation between serum Zn and BCAA levels in patients with HCV-related CLD, but that an inverse correlation between serum Zn and tyrosine levels did exist [51]. Therefore, an inverse correlation was found between serum zinc levels and the ratio of BCAA to tyrosine (BTR) in such patients.

Surprisingly, oral administration of BCAA granules caused a decrease in serum Zn levels of patients with liver cirrhosis [124]. The authors speculated that the protein synthesis was facilitated by the administration of BCAAs resulting in consumption of Zn, because Zn acts at multiple steps in amino acid- and insulin-regulated intracellular signaling pathways, including mTOR. It is of interest that the amount of urinary Zn secretion was related to the infusion of amino acid in patients who received parenteral nutrition [125]. Surprisingly, Zn bound to amino acids such as aspartate, cysteine and histidine showed the highest absorption concentration, followed by zinc chloride, sulfate and acetate, while zinc oxide showed the lowest bioavailability [116].

### 4.7. Zn and Hormones

It has been well established that Zn plays a fundamental role in the stability of IGF-1 transcripts [74]. Therefore, Zn deficiency eventually caused a decrease in the synthesis of IGF-1 in patients with HCV-related CLD [18]. Zn supplementation may increase the production of IGF-1 in the livers of patients with liver cirrhosis.

Zn seems to participate in modulating serum testosterone levels in normal men. Therefore, hypogonadism has been linked to Zn deficiency [126]. In contrast, Fuse and colleagues found a significant correlation between serum Zn and testosterone levels among infertile male patients [115]. Indeed, Zn supplementation facilitated the synthesis of testosterone in elderly men with marginal Zn deficiency [9].

## 5. Conclusions

This review complied overwhelming evidence that Zn deficiency is common in patients with chronic liver diseases such as chronic hepatitis and NASH, as well as liver cirrhosis. In such patients, Zn deficiency causes various types of metabolic abnormalities, including insulin resistance, hepatic steatosis, iron overload and hepatic encephalopathy. As expected, these metabolic abnormalities may be recovered by Zn supplementation. Further trials will be required to verify the beneficial effects of Zn administration on theses metabolic abnormalities especially in NASH patients.

## Figures and Tables

**Table 1:** Metabolic abnormalities causing zinc deficiency and metabolic abnormalities deriving from zinc deficiency.

Disease	Cause of Zinc Deficiency	Outcomes of Zinc Deficiency	References
Liver cirrhosis	Decrease in albumin synthesis and relative increase in α2-macroglobulin		[13,14]
	Decrease in Zn absorption from the small intestine		[15]
Hepatic encephalopathy	Liver cirrhosis	Decrease in ornithine transcarbamylase activity	[16]
Insulin resistance	Oxidative stress	Iron overload	[17]
		Decrease in free IGF-1 level	[18]
Hepatic steatosis	Oxidative stress	Enhancement of lipid peroxidation	[17]

IGF-1, insulin-like growth factor.

**Table 2:** Beneficial effects of zinc administration in patients with chronic liver disease.

Disease	Type of Zn Compound	Effect of Zinc Supplementation	Putative Mechanism	Reference
Hepatic encephalopathy	Zinc sulfate	Reduced ammonia levels	Recovery of OTC activity	[84]
	Zinc-hydrogen-asparate or zinc-histidine	Reduced ammonia levels	Recovery of OTC activity	[85]
	Zinc acetate	Reduced ammonia levels	Recovery of OTC activity	[86,87]
	Polaprezinc	Reduced ammonia levels	Recovery of OTC activity	[88]
Diabetes mellitus	Zinc sulfate	Reduced glucose levels	Improvement of insulin resistance	[89]
Chronic hepatitis	Polaprezinc	Reduced ALT levels	Improvement of iron overload	[90]
	Polaprezinc + IFN-based treatment	Higher rate of HCV eradication		[91]
	Polaprezinc + IFN-based treatment	Lower ALT levels		[92]
Wilson’s disease	Zinc acetate	Inhibition of Cu absorption	Increase in MT synthesis	[93]
HCC	Polaprezinc	Lower incidence of HCC development	Maintenance of hepatic reserve	[94]

OTC, ornithine transcarbamylase; HCV, hepatitis C virus; IFN, interferon; ALT, alanine aminotransferase; MT, metallothioneine; HCC, hepatocellular carcinoma.

**Table 3:** Interactions between zinc and other trace elements, vitamins, amino acids, or hormones in chronic liver disease.

Other Trace Elements, Vitamins, Amino Acids, or Hormones	Disease or Condition	Correlation	Reference
Copper	Liver cirrhosis	Increase in Cu/Zn ratio	[33]
	Liver cirrhosis	Increase in Cu/Zn ratio	[34]
	HCC	Increase in Cu/Zn ratio	[33]
	HCC	Increase in Cu/Zn ratio	[34]
	HCC	Increase in Cu/Zn ratio	[107]
	High dose of Zn prescription	Cu deficiency	[108]
Iron	HCV-related CLD	Inverse correlation	[17]
	HCV-related CLD with NAFLD	Inverse correlation	[109]
Selenium	Alcoholic liver disease, PBC, liver cirrhosis and autoimmune hepatitis	Positive correlation	[110]
	HCV-related CLD	Positive correlation	[111]
Vitamin A	Liver cirrhosis	Positive correlation	[112]
Retinol-Binding Protein	Liver cirrhosis	Positive correlation	
Vitamin D	Primary ovarian insufficiency	Positive correlation	[113]
	HCV-related CLD	No significant correlation	[114]
Branched-chain amino acids	HCV-related CLD	No significant correlation	[51]
Tyrosine	HCV-related CLD	Inverse correlation	[51]
IGF-1	HCV-related CLD	Positive correlation	[18]
Testosterone	Infertile males	Positive correlation	[115]

Cu, copper; Zn, zinc; HCC, hepatocellular carcinoma; HCV, hepatitis C virus; CLD, chronic liver disease; NAFLD, nonalcoholic fatty liver disease, PBC, primary biliary cholangitis; IGF-1, insulin-like growth factor-1.
